# Supplementary material for: Ferroptosis induction in host rice by endophyte OsiSh-2 is necessary for mutualism and disease resistance in symbiosis
Source: Nat Commun. 2024 Jun 12;15:5012. doi: 10.1038/s41467-024-49099-y (PMC11169551; doi:10.1038/s41467-024-49099-y)

Supplementary files

Ferroptosis induction in host rice by endophyte OsiSh-2 is necessary for mutualism and disease resistance in symbiosis

Xianqiu Xiong[1,2], Jing Zeng[1,2], Qing Ning[1], Heqin Liu[1], Zhigang Bu[1], Xuan Zhang[1], Jiarui Zeng[1], Rui Zhuo[1], Kunpeng Cui[1], Ziwei Qin[1], Yan Gao[1]✉, Xuanming Liu[1]✉, Yonghua Zhu[1,3]✉

[1]Hunan Province Key Laboratory of Plant Functional Genomics and Developmental Regulation, College of Biology, Hunan University, Changsha 410082, Hunan, PR China

[2]These authors contributed equally

[3]Lead Contact

✉email: yonghuaz@outlook.com, xml05@hnu.edu.cn, blessedgy@hnu.edu.cn

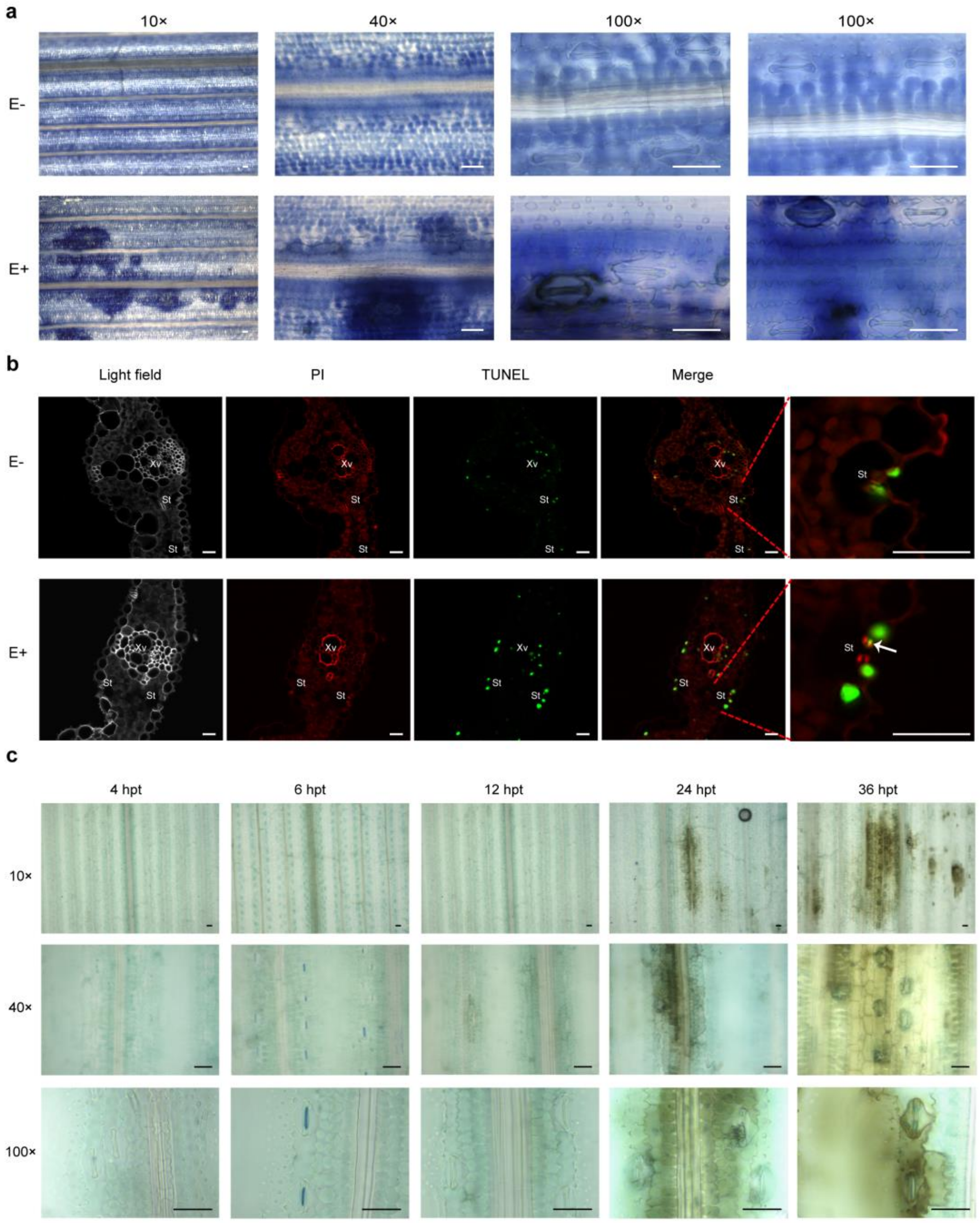

Supplementary Figure 1. *Streptomyces hygroscopicus* OsiSh-2 triggers cell death in and around stomata and adjacent mesophyll cells in rice leaves. a Microscopic magnified images of trypan blue staining (dark blue colour) show the cell death of E+ rice leaves at 24 hour post treatment (hpt). Scale bars: 20 μm. **b** Cell death and DNA damage assays by propidium iodide (PI, red signals) and terminal deoxynucleotidyl transferase-mediated dUTP-biotin nick-end labelling (TUNEL, green signals) staining, respectively, showed cell death in the stomata of E+ rice leaves at 6 hpt. One representative example out of three independent image is shown. Xv, xylem vessel element; St, stomata. Scale bars: 20 μm. **c** Microscopic magnified images of Prussian blue staining show the change in Fe^{3+} accumulation in rice leaves at 4-36 hpt. Scale bars: 20 μm. The images shown are

representative of rice leaf samples in different treatments. All experiments were repeated independently three times with similar results. Source data are provided as the Source Data file.

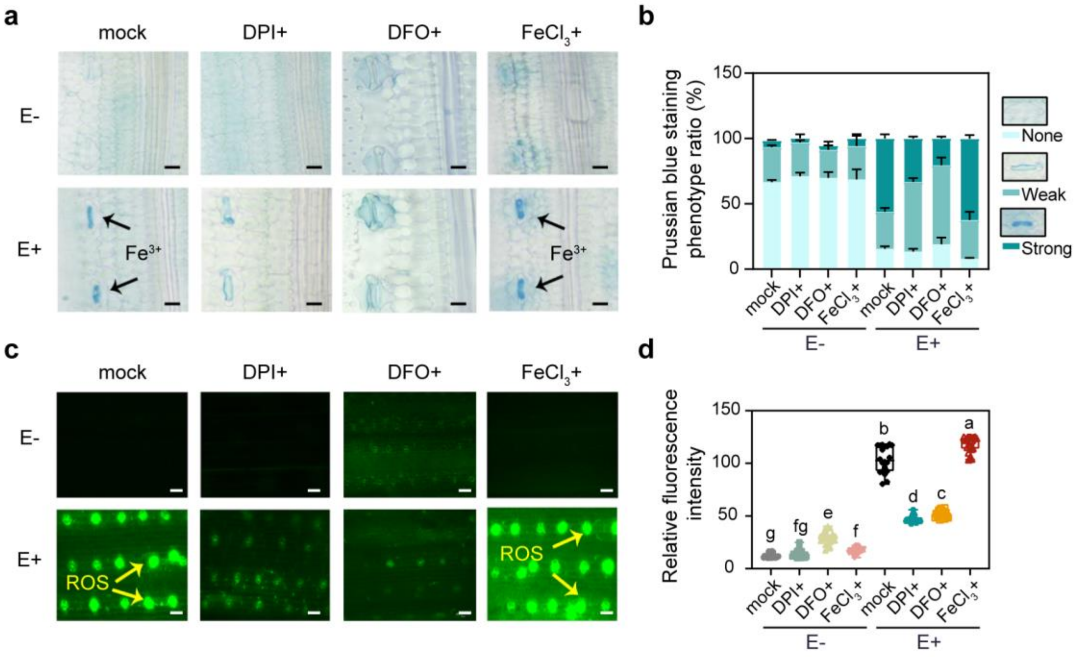

Supplementary Figure 2. Ferroptosis inhibitors (DPI and DFO) and inducer ($FeCl_3$) influence the Fe^{3+} and ROS accumulation in rice leaves. a Prussian blue staining shows the accumulation of Fe^{3+} in E- and E+ rice leaves treated with water (mock), diphenyleneiodonium (DPI+), deferoxamine (DFO+), and $FeCl_3$ ($FeCl_3$+) at 6 hpt. Scale bars: 10 μm. The black arrows indicate Fe^{3+} accumulation. **b** Relative Fe^{3+} accumulation is expressed as the Prussian blue staining phenotype ratio (%). Ratios indicate the proportions of designated staining phenotypes. Error bars indicate the mean ± SDs (n=3 for each). **c** CM-H_2DCFDA staining shows the accumulation of reactive oxygen species (ROS, green fluorescence) in rice samples same as (**a**), at 6 hpt. Scale bars: 20 μm. The yellow arrows indicate ROS bursts. **d** Relative ROS accumulation is expressed as the relative fluorescence intensity of CM-H_2DCFDA-stained rice cells. The relative fluorescence intensity of ROS was calculated via ImageJ. Experimental repeats are displayed as box plots with individual data points. The error bars represent maximum and minimum values. Middle horizontal bars of boxplots represent the median, and the bottom and top represent the 25th and 75th percentiles (n=20 for each).

The bars with different letters are significantly different (ANOVA, $P < 0.05$) according to Duncan's multiple-range test. The images shown are representative of the rice leaf samples in different treatments. All experiments were repeated independently three times with similar results. Source data are provided as the Source Data file.

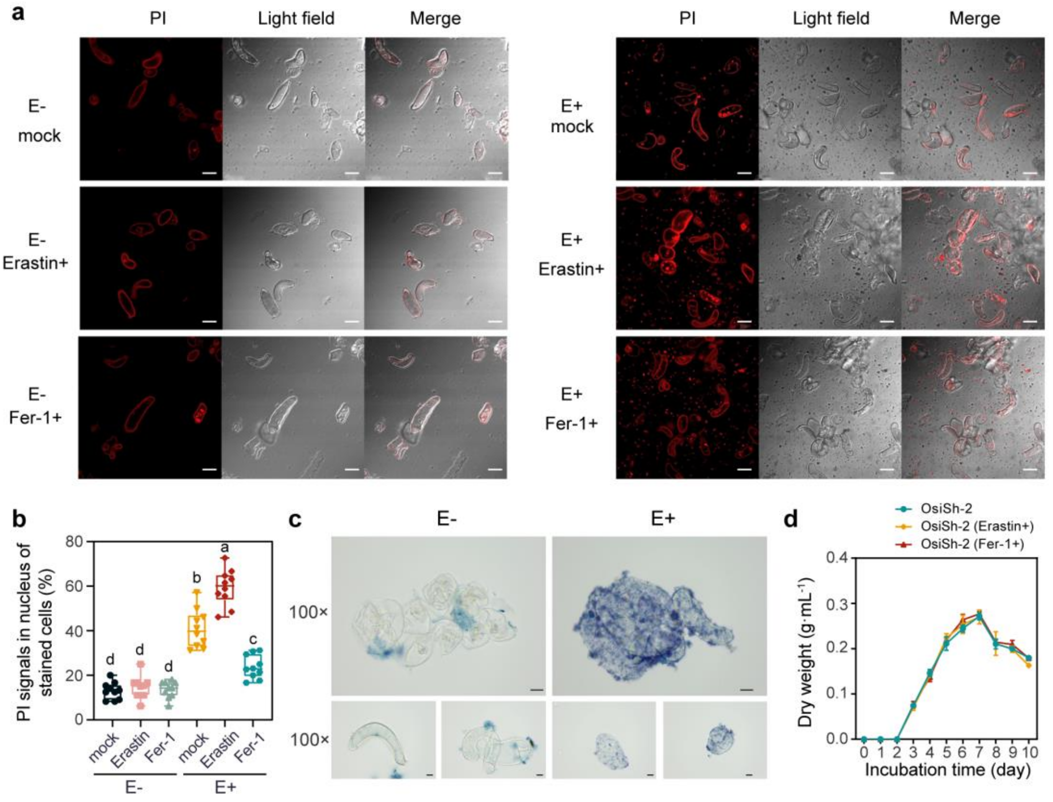

Supplementary Figure 3. ***Streptomyces hygroscopicus*** **OsiSh-2 triggers the death of rice suspension cells. a** Propidium iodide (PI) staining observe the cell viability of E- and E+ rice suspension cells treated with water (mock), erastin (Erastin+), and Fer-1 (Fer-1+) at 6 hpt by using a laser scanning confocal microscope. Scale bars: 30 μm. The images shown are representative of rice suspension cells in different treatments. **b** PI signals in nuclei of stained cells show the suspension cell death rate by propidium iodide (PI) staining. Experimental repeats are displayed as box plots with individual data points. The error bars represent maximum and minimum values. Middle horizontal bars of boxplots represent the median, and the bottom and top represent the 25th and 75th percentiles (n=10 for each). The bars with different letters are significantly different (ANOVA, $P < 0.05$) according to Duncan's multiple-range test. **c** Prussian blue staining (blue colour) shows the accumulation of Fe^{3+} in E- and E+ rice suspension cells at 6 hpt by using an optical microscope. Scale bars: 20 μm. **d** The growth curve indicated by the dry weight ($g \cdot mL^{-1}$) of OsiSh-2, OsiSh-2 (Erastin+), and OsiSh-2 (Fer-1+) in ISP2 liquid medium for 0-10 days. Error bars indicate the mean ± SDs (n=3). All experiments were repeated independently three times with similar results. Source data are provided as the Source Data file.

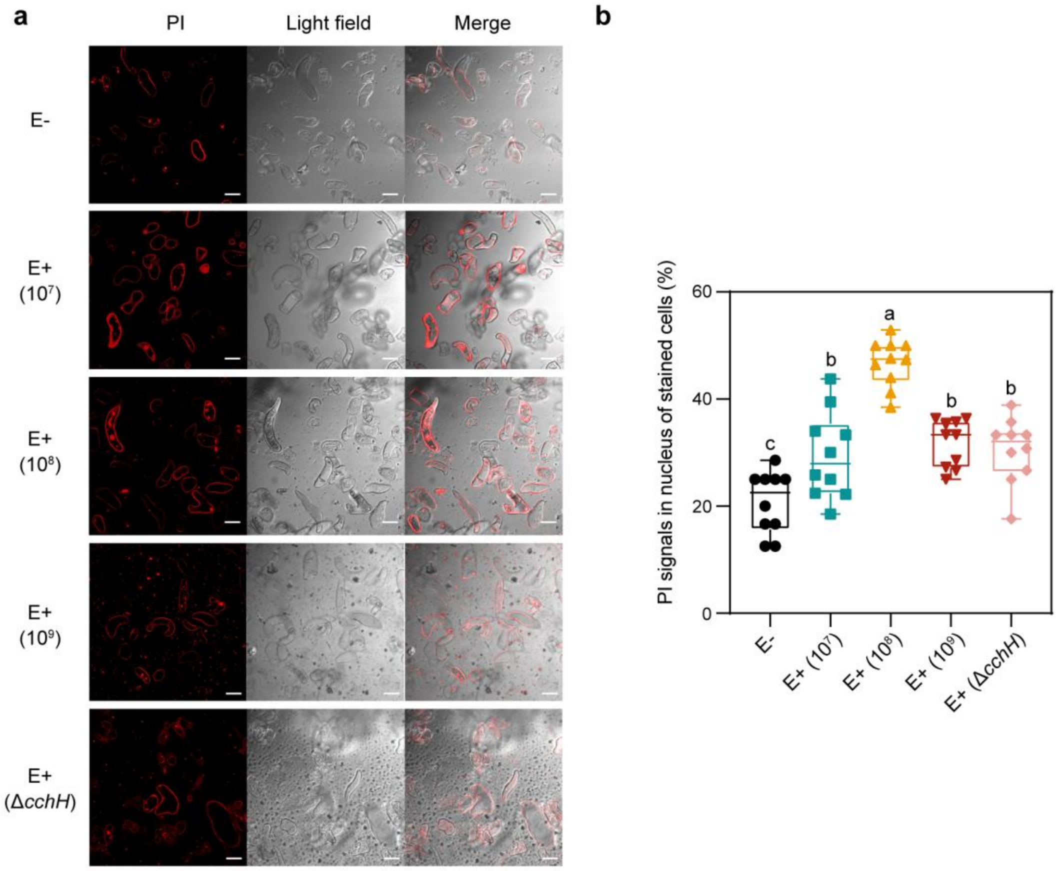

Supplementary Figure 4. *Streptomyces hygroscopicus* OsiSh-2 triggered rice cell death depends on its appropriate concentration and normal siderophore production capability. a Propidium iodide (PI) staining observe the viability of rice suspension cells treated with OsiSh-2 ($10^{7/8/9}$ spore mL^{-1}), and Δ*cchH* (*ShCchH* knockout mutant strains of OsiSh-2) at 6 hpt by using a laser scanning confocal microscope. Scale bars: 30 μm. The images shown are representative of rice suspension cells in different treatments. **b** PI signals in nuclei of stained cells show the suspension cell death rate by propidium iodide (PI) staining. Experimental repeats are displayed as box plots with individual data points. The error bars represent maximum and minimum values. Middle horizontal bars of boxplots represent the median, and the bottom and top represent the 25th and 75th percentiles (n=10 for each). The bars with different letters are significantly different (ANOVA, $P < 0.05$) according to Duncan's multiple-range test. All experiments were repeated independently three times with similar results. Source data are provided as the Source Data file.

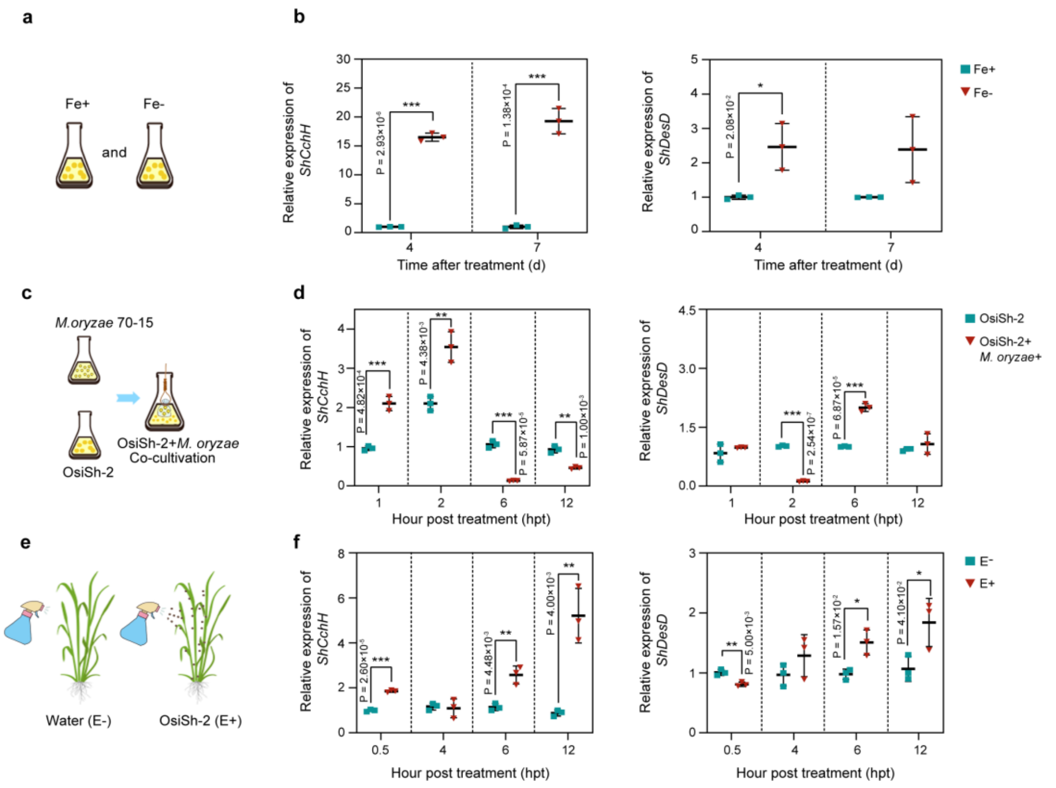

Supplementary Figure 5. Siderophores produced by *Streptomyces hygroscopicus* OsiSh-2 participate in the response to environmental changes, including iron deficiency, *Magnaporthe oryzae* stress, and colonization in host rice. a Schematic of OsiSh-2 when cultured in iron sufficient (Fe+) and iron deficient (Fe-) media. **b** The transcript levels of *ShCchH* (a core synthesis-related gene of the siderophore COE) and *ShDesD* (a core synthesis-related gene of the siderophore DFO) were analysed via qRT–PCR when OsiSh-2 was cultured in Fe+ and Fe- media for 4 and 7 days. Error bars indicate the mean ± SDs (n=3). **c** Schematic of OsiSh-2 when cocultivated with *M. oryzae* 70-15. Both OsiSh-2 and *M. oryzae* 70-15 were in the logarithmic phase before cocultivation. **d** The transcript levels of *ShCchH* and *ShDesD* were analysed via qRT–PCR at the indicated times after OsiSh-2 was cocultivated with *M. oryzae* 70-15 or not. Others are as in (b). **e** Schematic of OsiSh-2 when evenly sprayed on the surface of rice leaves. **f** The transcript levels of *ShCchH* and *ShDesD* were analysed via qRT–PCR at the indicated hours post treatment in water treated (E-) and OsiSh-2 treated (E+) rice. Others are as in (b).

The bars with asterisks are significantly different as determined by the Tukey–Kramer test (*, $P < 0.05$; **, $P < 0.01$; ***, $P < 0.001$). All experiments were repeated independently three times with similar results. Source data are provided as the Source Data file.

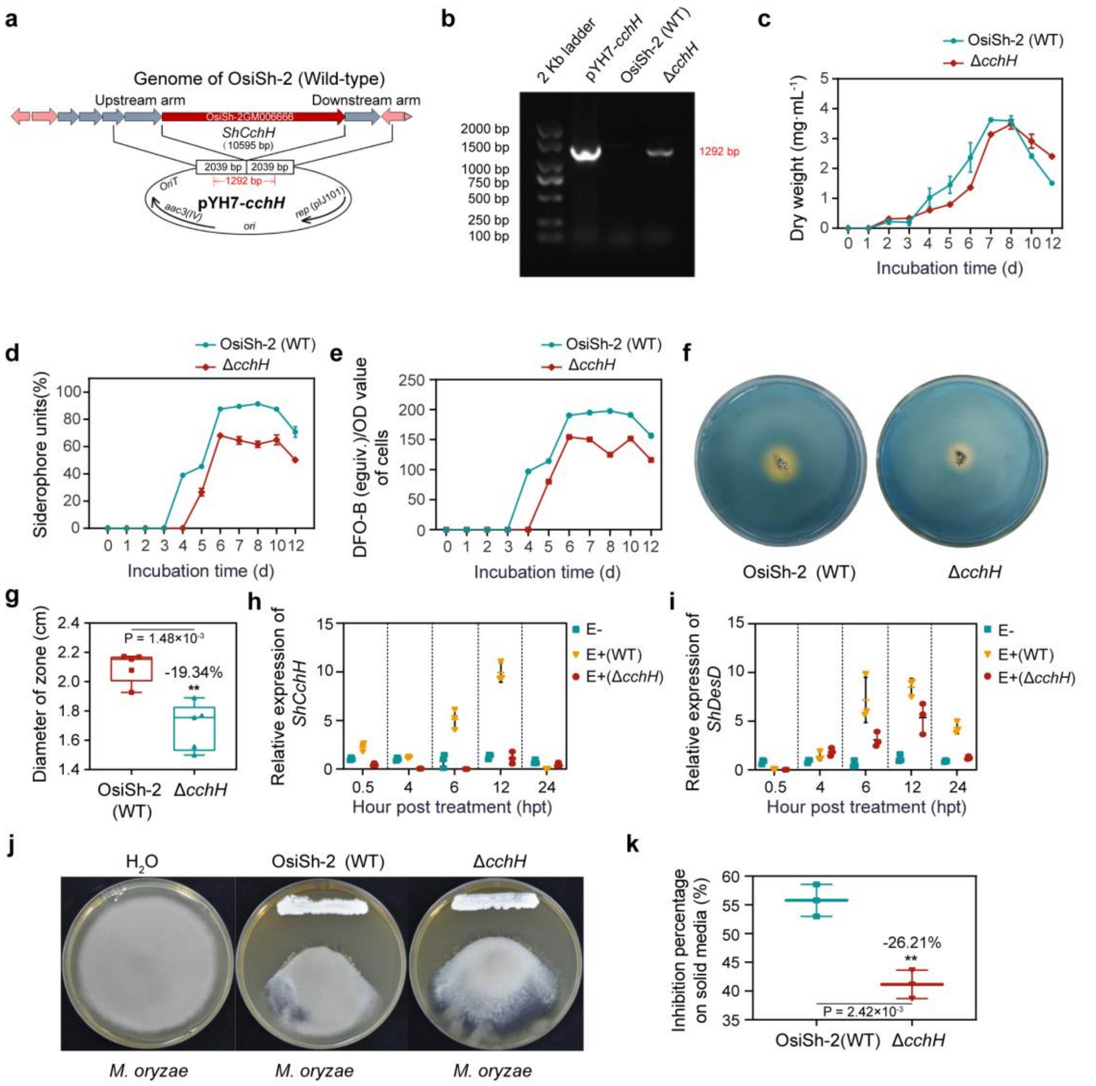

Supplementary Figure 6. Characterization of *Streptomyces hygroscopicus* OsiSh-2 (wild-type, WT) and Δ*cchH* (*ShCchH* knockout mutant strains of OsiSh-2). a Schematic of the test primer sites in the recombinant plasmid pYH7-*cchH*. **b** Image verifying the PCR results of Δ*cchH* via test primers. **c** Growth curve of OsiSh-2 (WT) and Δ*cchH* in ISP2 solid media for 0-12 days. Error bars indicate the mean ± SDs (n=3). **d, e** Siderophore production of OsiSh-2 (WT) and Δ*cchH* in siderophore-producing liquid media for 0-12 days. Siderophore units (%) (**d**) and DFO-B (eguiv.)/OD value (**e**) were determined by two different calculation methods. Others are as in (c). **f** Siderophore production by OsiSh-2 (WT) and Δ*cchH* on chrome azurol S (CAS) agar plates evident by orange zones. **g** The activity of siderophores was indicated by the diameter of the orange zone in CAS plates. Experimental repeats are displayed as box plots with individual data points. The error bars represent maximum and minimum values. Middle horizontal bars of boxplots represent the median, and the bottom and top represent the 25th and 75th percentiles (n=5 for each). **h, i** The transcript levels of *ShCchH* (**h**) and *ShDesD* (**i**) were analysed via qRT–PCR at the indicated hours

post treatment in E+ (WT) and E+ (Δ*cchH*) rice. Error bars indicate the mean ± SDs (n=3). **j** Antagonism of OsiSh-2 (WT) and Δ*cchH* against *M. oryzae* on potato dextrose agar (PDA) medium. The distance between *M. oryzae* and OsiSh-2 (WT) or Δ*cchH* was 30 mm. **k** The growth inhibition percentage of *M. oryzae* by OsiSh-2 (WT) and Δ*cchH*. Others are as in (c).

The bars with asterisks are significantly different as determined by the Tukey–Kramer test (**, $P < 0.01$). The percentage changes followed by "+" or "−" above the bars were calculated by using the following formula: percent change = [(value of OsiSh-2 (WT) or Δ*cchH* -treated group) − (value of H_2O -treated group)]/(value of H_2O -treated group) × 100%. All experiments were repeated independently three times with similar results. Source data are provided as the Source Data file.

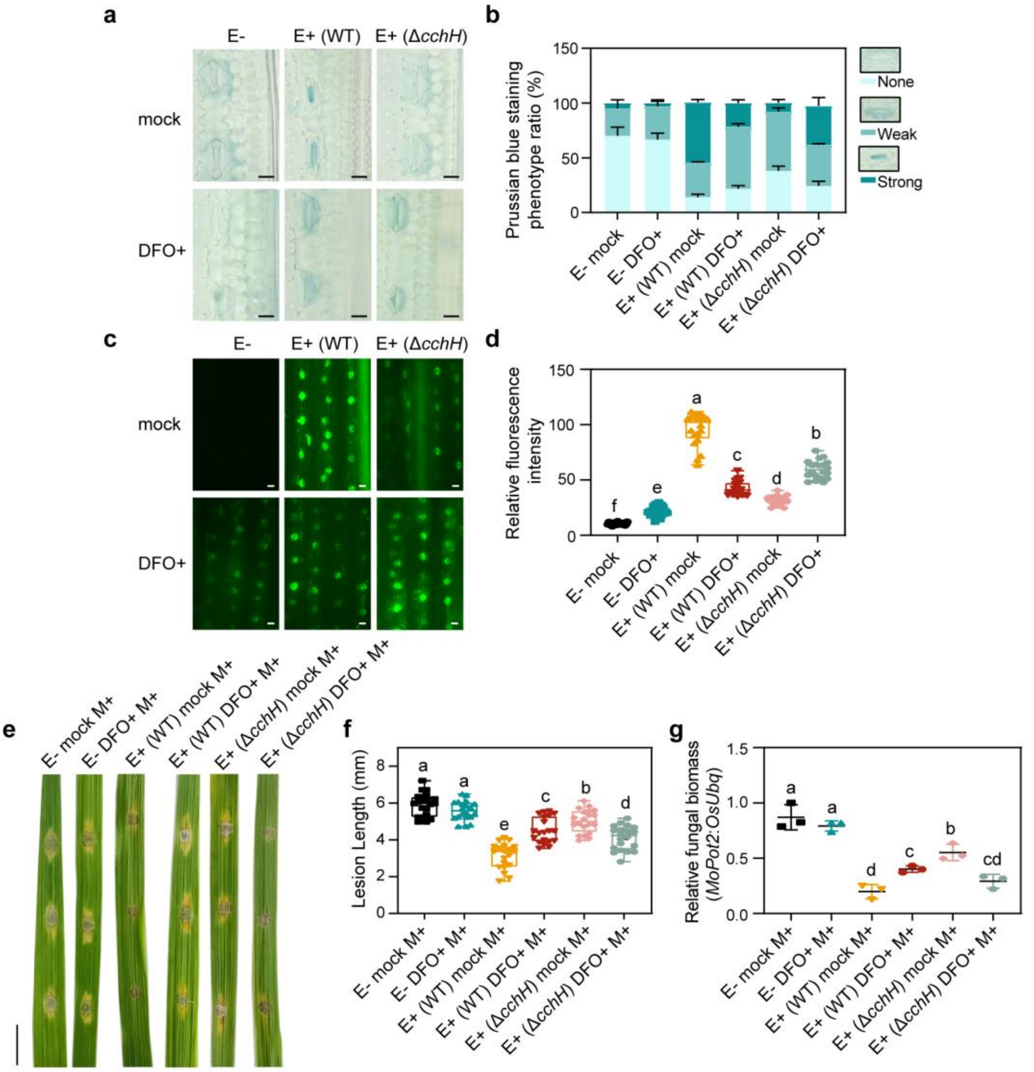

Supplementary Figure 7. Siderophore deferoxamine (DFO) can partially rescue the induction of ferroptosis in Δ*cchH* treated rice. a Prussian blue staining (blue colour) shows the accumulation of ferric ions (Fe^{3+}) in E-, E+ (WT), and E+ (Δ*cchH*) rice leaves treated with DFO (DFO+) and water (mock) at 6 hpt. Scale bars: 10 μm. **b** Relative Fe^{3+} accumulation is expressed as the Prussian blue staining phenotype ratio (%). Ratios indicate the proportions of designated staining phenotypes. Error bars indicate the mean ± SDs (n=3). **c** CM-H_2DCFDA staining shows the accumulation of ROS (green fluorescence) in rice leaves same as (**a**), at 6 hpt. Scale bars: 20 μm. **d** Relative ROS accumulation is expressed as relative fluorescence intensity of CM-H_2DCFDA-stained rice cells. The relative fluorescence intensity of ROS was calculated via ImageJ. Experimental repeats are displayed as box plots with individual data points. The error bars represent maximum and minimum values. Middle horizontal bars of boxplots represent the median, and the bottom and top represent the 25th and 75th percentiles (n=20 for each). **e** Images of blast lesions on detached rice leaf segments at 5 days post-infection (dpi) of *M. oryzae* at a concentration of 10^5 conidia mL^{-1} (M+) in rice leaves

same as **(a)**. Scale bars: 1.0 cm. **f** The lesion length of detached rice leaves was measured at 5 dpi via ImageJ in rice leaves. Others are as in (d). **g** The relative fungal biomass of *M. oryzae* was calculated by DNA-based qRT–PCR at 5 days post infection in rice leaves. Error bars indicate the mean ± SDs (n=3).

The bars with different letters are significantly different (ANOVA, $P < 0.05$) according to Duncan's multiple-range test. All experiments were repeated independently three times with similar results. Source data are provided as the Source Data file.

Supplementary Table 1. Primers for constructs and qRT-PCR in this study

Prime name	Primer sequence (5'-3')	Purpose
ShCchH-test-F	CATGGTGAGCATCCTGACGCAGGACACGCAT GTGTTCTCC	Test for *ShCchH*-knock out strain
ShCchH-test-R	GTGCTTCGCCTCCGTGCTGACCGCACGGTAG GACCGGAAC	Test for *ShCchH*-knock out strain
ShCchH-L-F	CGCCATATGCAGCTGTTCGGCAACCTGGACG CCGTGCGG	Knock out of *ShCchH*
ShCchH-L-R	CCGGAATTCACGAGCGCCCGGTTCCACCATC GAGGACTC	Knock out of *ShCchH*
ShCchH-R-F	CCGGAATTCGTCCTCGATGACAACTCCTGAC CCGCGGCG	Knock out of *ShCchH*
ShCchH-R-R	CCCAAGCTTGCCGGACCCCTTGGGCGGCCCG GTCGGCGG	Knock out of *ShCchH*
OsLOX2-F	AGATGAGGCGCGTGATGAC	qRT-PCR
OsLOX2-R	CATGGAAGTCGAGCATGAACA	qRT-PCR
OsWRKY70-F	CCGCTGCTGTTTTGATCATCT	qRT-PCR
OsWRKY70-R	GGAGCTAAGCTAACTCACTCCACA	qRT-PCR
OsPBZ1-F	GTGGGAAGCACATACAAGACC	qRT-PCR
OsPBZ1-R	AGGGTGAGCGACGAGGTAG	qRT-PCR
OsAOS2-F	TACCAGCCGTGCGCCACCAG	qRT-PCR
OsAOS2-R	AGGACGGAGCTGGTTGAGTGG	qRT-PCR
OsPR1a-F	TCGTATGCTATGCTACGTGTTT	qRT-PCR
OsPR1a-R	CACTAAGCAAATACGGCTGACA	qRT-PCR
OsActin-F	GAGTATGATGAGTCGGGTCCAG	qRT-PCR
OsActin-R	ACACCAACAATCCCAAACAGAG	qRT-PCR
OsYSL15-F	GGATTGCAGAAATAAACAGTGATG	qRT-PCR
OsYSL15-R	TGCCAAACTAAACAATTCTCAA	qRT-PCR
OsIRO2-F	GGCTACCTGCATCAATGAC	qRT-PCR
OsIRO2-R	GCTTTGTTCCTGACGACTTT	qRT-PCR
MoPot2-F	ACGACCCGTCTTTACTTATTTGG	qRT-PCR
MoPot2-R	AAGTAGCGTTGGTTTTGTTGGAT	qRT-PCR
OsUbq-F	TTCTGGTCCTTCCACTTTCAG	qRT-PCR
OsUbq-R	ACGATTGATTTAACCAGTCCATGA	qRT-PCR
ShDesD-F	GCCGACTACCACCTGCTGC	qRT-PCR
ShDesD-R	GCGGTTTTCACATAGTGCTTGG	qRT-PCR
ShCchH-F	TGCGGGAACACCAGAGCG	qRT-PCR
ShCchH-R	TCAACGGGTAGTGCGGAGC	qRT-PCR
ShRpoA-F	GCAAGGGCAAGCTGGAGATG	qRT-PCR
ShRpoA-R	ATGGAGTCGACCGGGATACG	qRT-PCR

Supplementary Table 2. *Escherichia coli* strains used in the study

Strain	Relevant genotype and characteristics	Source
DH5α	*supE44 hsdR17 recAl endAl gyrA19 thi-1 relAl △lacU169 (80lacZ△M15)*	(1)
ET12567/pUZ8002	rec^F *dam dcm* Cml^R Str^R Tet^R Km^R	(2)

1. Grant, S.G. et al. Differential plasmid rescue from transgenic mouse DNAs into *Escherichia coli* methylation-restriction mutants. *Proc Natl Acad Sci U S A*. **87**, 4645-9 (1990).
2. MacNeil, D. J. et al. Analysis of *Streptomyces avermitilis* genes required for avermectin biosynthesis utilizing a novel integration vector. *Gene*. **111**, 61–68 (1992).

Supplementary Text 1. The sequencing result of identified positive Δ*cchH* mutant strain

Detection primers for the identification of Δ*cchH* (*ShCchH* knockout mutant strain of OsiSh-2):
ShCchH-test-F: CATGGTGAGCATCCTGACGCAGGACACGCATGTGTTCTCC 40 bp
ShCchH-test-R: GTGCTTCGCCTCCGTGCTGACCGCACGGTAGGACCGGAAC 40 bp

The test primers amplified gene fragment of *ShCchH* (1292 bp):
CATGGTGAGCATCCTGACGCAGGACACGCATGTGTTCTCCGGTCCGCTCGCCGACGAC
CTGCGGCTGGCCGCGCCGGAGGCGACCGACGCCGAGCTGATGGACGCGTTGCGTAGG
GTCGGCGCCGACGGGTGGGTCGACGCGCTGCCCGACAGGCTGAACACCATGGTCGGC
GAGGGCGGAGAGCGGCTGGACGTCACCAAGGTCGCCCAGATCGCCCTGGCCCGGCTG
GTGCTGGCCCGGACGCCGGTGGTGGTGCTCGACGAGTCGACCGCGGAGGCCGGCAGC
GAGGGCGCCGCCGAGCTGGAGCGGGCCGTGCTGGCCGCGTGCTCGGGCCGGACCACG
TTGTTCGTGGCGCACCGGCTGACCCAGGCGATGGCGGCGGACCGGATCGCCGTGCTG
GATACCGGACGCGTGGTGGAGCAGGGAACCCACGAGGAGCTGGTGGCCCTGGGCGGC
CGGTACGCGCGACTGTGGCGGGCCTGGCGAGAAGGCAGTTAGCCGGCCCTGATTCAG
ATGAGCTTTTCATGACGCGGAAGGGATGCTGAGTCCTCGATGGTGGAACCGGGCGCTC
GTGTCCTCGATGACAACTCCTGACCCGCGGCGGCCCCGACCGGGGTCCGCCATCCTCC
GGACCGCACTGCGCCGCAACACAGGCGCCATGGCCTGGGGCACCGTCCTCATGGGCC
TGTACCAGGCGGGTGAGACGGCCTTCCCCATCGCGCTCGGCCTGATCGTCGAGCACAC
GATGCGTCACCGGAGCCTCGGCGCGCTGGGCCTGTCGATCGCCGCACTGGCGGTGATC
ATCACGACGGTGTCGCTGTCGTGGCGGTTCGGGATGCGCATCCTCCAGAAGGCCAACA
CGACCGAGGCACACCGCTGGCGGGTGAAGGTCGCGGCCTGTGGCCTTCAGCCGGTGG
CCAGGGACATGGACCTCAAGTCCGGCGAGGTGCTGACCATCGCCACCGAGGACGCCG
ACCAGACCGCCGACATCATCGAGGTGGTGCCGCTGCTGATCAGCTCGCTGGTCGCGGT
GCTGGTCGCGGCGGTCGCCCTCGGCATGGCCGACGTGCGGCTCGGCCTGCTGGTGATC
GTGGGAACCGTCGGGATCCTGTCGGCCCTGAGCGTGCTGTCCAGGCGGATCGGCTCCA
GCACCCGCGAACAGCAGGCCCGGGTGGCACGGGCGGGCGCGAAGGTCGCCGACCTG
ATCACCGGGCTGCGCCCGCTGCACGGCTTCGGCGGCAACCACGCGGCGTTCCGGTCCT
ACCGTGCGGTCAGCACGGAGGCGAAGCAC

The sequencing result of amplified gene fragment of *ShCchH*:
CAAAAAAGTTCTAGTTCATCTCTATCTCCACTACCTGCGGCTGGCCGCGCCGGAGGCG
ACCGACGCCGAGCTGATGGACGCGTTGCGTAGGGTCGGCGCCGACGGGTGGGTCGAC
GCGCTGCCCGACAGGCTGAACACCATGGTCGGCGAGGGCGGAGAGCGGCTGGACGTC
ACCAAGGTCGCCCAGATCGCCCTGGCCCGGCTGGTGCTGGCCCGGACGCCGGTGGTG
GTGCTCGACGAGTCGACCGCGGAGGCCGGCAGCGAGGGCGCCGCCGAGCTGGAGCG
GGCCGTGCTGGCCGCGTGCTCGGGCCGGACCACGTTGTTCGTGGCGCACCGGCTGAC
CCAGGCGATGGCGGCGGACCGGATCGCCGTGCTGGATACCGGACGCGTGGTGGAGCA
GGGAACCCACGAGGAGCTGGTGGCCCTGGGCGGCCGGTACGCGCGACTGTGGCGGGC
CTGGCGAGAAGGCAGTTAGCCGGCCCTGATTCAGATGAGCTTTTCATGACGCGGAAGG
GATGCTGAGTCCTCGATGGTGGAACCGGGCGCTCGTGAATTCCGGAATTCGTCCTCGA
TGACAACCCTGACCCGCGGCGGCCCCGACCGGGGTCCGCCATCCTCCGGACCGCACT
GCGCCGCAACACAGGCGCCATGGCCCTGGGGCACCGTCCTCATGGGCCTGTACCAGGC

GGGTGAGACGGCCTTCCCCATCGCGCTCGGCCTGATCGTCGAGCACACGATGCGTCAC
CGGGAGCCTCGSSSCGCTGGGCCTGTCGATCGCCGCACTGGCGGTGATCATCACGACG
GTGTCGCTGTCGTGGCGGTTCGGGATGCGCATCCTCCAGAAGGCCAACACGACCGAG
GCACACCGCTGGCGGGTGAAGGTCGCGGCCTGTGGCCTTCAGCCGGTGGCCAGGGAC
ATGGACCTCAAGTCCGGCGAGGTGCTGACCATCGCCACCGAGGACGCCGACCAGACC
GCCGACATCATCGAGGTGGTGCCGCTGCTGATCAGCTCGCTGGTCGCGGTGCTGGTCG
CGGCGGTCGCCCTCGGCATGGCCGACGTGCGGCTCGGCCTGCTGGTGATCGTGGGAAC
CGTCGGGATCCTGTCGGCCCTGAGCGTGCTGTCCAGGCGGATCGGCTCCAGCACCCGC
GAACAGCAGGCCCGGGTGGCACGGGCGGGCGCGAAGGTCGCCGACCTGATCACCGG
GCTGCGCCCGCTGCACGGCTTCGGCGGCAACCACGCGGCAGTGCTATGACCG

Sequence alignment of amplified gene fragment and the sequencing gene fragment of *ShCchH* (92.51% similarity):

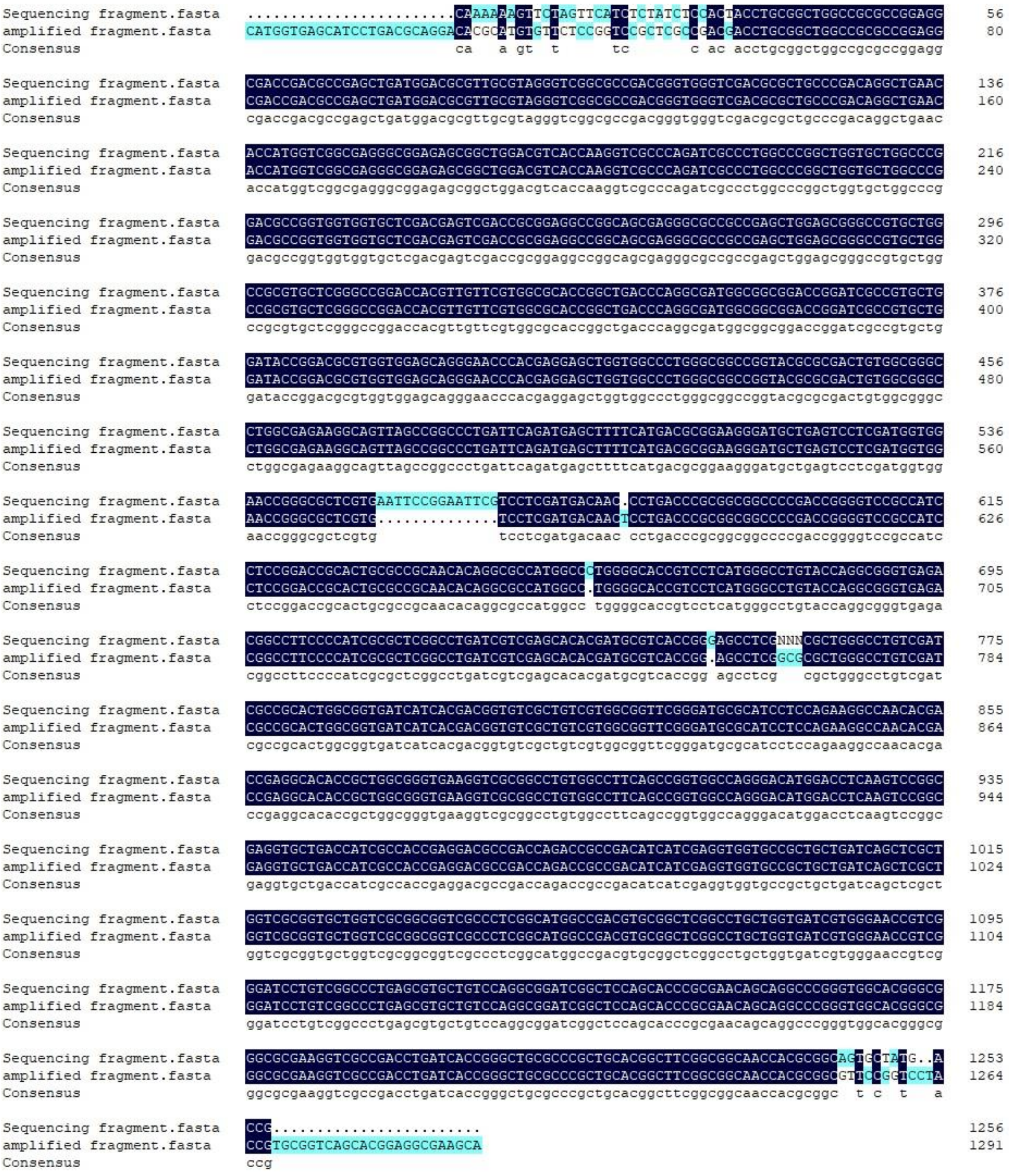

Supplement: Supplementary file 1 — Supplementary Information [file 41467_2024_49099_MOESM1_ESM.pdf]
